# *“You Want to Eat Healthy, Especially When You're Pregnant. But Sometimes, It's Just Not Possible”*: Perceptions of Facilitators and Barriers to Healthy Food and Diet Practices During Pre‐Conception and Pregnancy

**DOI:** 10.1111/jhn.70122

**Published:** 2025-09-07

**Authors:** Jane McClinchy, Rosalind Fallaize, Kelly Parsons, Lisa Whiting

**Affiliations:** ^1^ School of Health, Medicine and Life Sciences University of Hertfordshire Hatfield UK; ^2^ MRC (Medical Research Council) Epidemiology Unit University of Cambridge Cambridge UK

**Keywords:** co‐creation, diet, nutrition, pre‐conception, pregnancy, qualitative

## Abstract

**Background:**

Evidence suggests that women should eat a healthy diet during pre‐conception and pregnancy as this benefits their own health as well as reducing the risk of non‐communicable diseases in offspring (such as obesity, diabetes, hypertension, cardiovascular and mental health problems); however, previous work indicates that the recommendations are not being followed. This study aimed to understand: the facilitators and barriers to healthy food and diet practices during pre‐conception and pregnancy; how these barriers could be addressed, and the changes required to facilitate good food practices.

**Methods:**

The research used a qualitative approach; five online focus groups were undertaken with 19 women living across the UK who were trying to conceive, pregnant or had babies under 6‐months old. Data were thematically analysed.

**Results:**

The findings revealed three main themes (Challenges of trying to eat healthily; Facilitators to eating healthily; Changes required) and six subthemes (Mothers' load; Body sabotage; Food environment; Information not individualised; Planning skills; Family support; Co‐creation and investment for the future; Access to professional advice). Participants spoke of internal factors (such as tiredness and nausea) and external influences (e.g., their financial situation) that impacted on their ability to eat healthily. They identified the need to access more effective professional advice.

**Conclusions:**

This unique study demonstrated a need for clear, consistent, engaging and culturally appropriate dietary information, as well as access to professionals (such as nutritionists and dietitians) who can give both generic and tailored advice to those trying to conceive and those who are already pregnant.

## Summary

- It is important to acknowledge the impact that pregnancy can have on the ability to eat a healthy diet—this includes the side effects such as nausea and tiredness, but also other external factors such as affordability and partner support.
- Expectant parents/parents, especially mothers, are actively seeking information.
- Expectant parents/parents want access to healthcare professionals who can provide both generic as well as individually focussed advice.

## 1 Introduction/Background

Dietary advice for pregnancy in the UK focuses on eating a healthy balanced diet, (incorporating guidance on eating well if women are vegetarian or vegan) and on foods to avoid [1, 2, 3, 4, 5]. Poor nutritional intake during the pre‐conception period and throughout pregnancy can have a negative impact on women's fertility, pregnancy, and birth outcomes [6, 7, 8] and can increase the risk of non‐communicable diseases in offspring [9, 10].

There are several population‐based initiatives in the UK that have the potential to help pregnant women consume a healthy diet. In England, Northern Ireland, and Wales, the Healthy Start Scheme supports households (receiving specific benefits or earning £408 or less per month after tax) that include women > 10 weeks pregnant or children under four in England [11, 12, 13] with a prepaid card that can be used to buy fruit, vegetables, milk or infant formula based on cow's milk. Scotland has its own equivalent scheme called Best Start Foods [12]. While the Healthy Start Schemes have been found to be effective [14], although available to all those pregnant under 18, they are limited by their restrictive eligibility criteria [15], the declining value of the vouchers in relation to the rising cost of food and access to shops that employ the scheme [16]. In England, Family Hubs—local support centres for families with children may offer a source of nutrition information given by healthcare professionals (HCPs) in contact with pregnant women and young families, as recommended by the First Steps Nutrition Trust [17]. An interim evaluation of the Family Hubs initiative found that both health professionals and families preferred this ‘drop in’ facility rather than a structured programme [18]. The Office for Health Improvement and Disparities (OHID) (previously Public Health England) [10, 19] promotes a life course approach to health and wellbeing aimed at ‘making every contact count’ between HCPs and the public [10]. The Obesity Health Alliance [20] proposes ‘touch points’ where this could take place, stating that it should include family planning clinics, health‐visitor contacts, and the 6‐week postnatal check.

NICE guidance on maternal and child nutrition [5] recommends that health professionals should be facilitated to provide healthy eating advice during pregnancy. The information should be non‐judgemental, tailored, and evidence‐based considering service user circumstance and enable them to gain the skills needed to be able to follow a healthy diet. NICE [21] advises a first midwife booking appointment by week 10 of the pregnancy followed by 6–9 additional appointments with either a midwife or doctor, with nutrition information being provided at each of these [22]. However, there are limitations to relying on these appointments to deliver nutritional advice; although pregnancy offers a ‘teachable moment’ [23] and women are motivated to adopt healthy behaviours once pregnant [22], only 53.9% of pregnant women attend a booking appointment within 10 weeks [24] and so the information may be too late to have a positive impact on pregnancy outcomes [7, 25]. The nutritional advice that midwives can provide may also be limited by lack of knowledge and available time [26, 27, 28].

OHID, (the UK Government's public health agency) suggests that all HCPs have a role to play in giving nutritional advice to pregnant women and that a collaborative approach is needed [19]. Whilst dietitians and nutritionists are the only HCPs who are specifically trained in this area, they are not routinely employed by the NHS in the UK to see women in pregnancy [29, 30]. Evidence suggests that midwives are only able to provide limited dietary advice to pregnant women [26, 28] and much of this is focused on food safety or managing medical conditions such as hyperemesis gravidarum or diabetes [28, 29]. It has been suggested that dietitians could help in resource development, training, and antenatal appointments [26]. Grenier, Atkinson, Mottola et al. [31] found that pregnant women thought that nutritionists should be a standard part of pregnancy care.

Despite policy, guidance and initiatives, nutritional recommendations in pregnancy are not being met and HCPs may be struggling to support the delivery of nutritional advice [6, 28, 32].

Our research team was commissioned by The Food Foundation to explore:

- The facilitators and barriers to healthy food and diet practices during pre‐conception and pregnancy.
- How the barrier(s) to healthy diets at this life stage could be addressed.
- The changes required to facilitate good food practices.

## 2 Methods

The study used a qualitative approach. Qualitative research helps explain why people behave in particular ways and is especially useful for understanding food practices [33]. Reporting of the study was guided by the consolidated criteria for reporting qualitative research (COREQ) 32‐item checklist for interviews and focus groups [34].

Purposive sampling is used to recruit participants who fulfil a particular inclusion criteria [35]. In our study, the participants were parents/expectant parents and those planning a pregnancy from across the four nations of the UK. Recruitment was via a range of relevant private Facebook pages.

Focus groups were used because they can facilitate insight into the participants' experiences of their day to day lives. They make use of group dynamics building on each other's stories and inspire participants who may be reluctant to contribute and who feel more confident to participate in group discussions [36, 37, 38].

The aim was to recruit between 5 and 7 participants to each focus group to facilitate moderation and rich data collection [39, 40], however it was decided, in advance, to go ahead with a planned session should attendance not reach 5 participants. To achieve theoretical saturation [38] focus groups were scheduled to facilitate attendance and recruitment of participants across the four countries.

As some COVID‐19 pandemic precautions were still in place, data collection was undertaken online. Although research suggests that responses in online focus groups may be shorter and less complex than a face to face session [41] they have been found to enable the collection of rich data and can be successfully used as an option where data collection opportunities are limited [42].

### 2.1 Data Collection

Focus groups took place February‐March 2022 via Zoom (Zoom Video Communications Inc.) and were digitally recorded. Three focus groups, ranging from 3 to 7 participants and 2 very small focus groups (VSFG) [37] were undertaken with parents/expectant parents. All authors contributed to development of the guidelines, topic guide and ‘prompt’ questions (although not piloted before use). Focus groups were led by RF and JM (both female), who introduced themselves before each one and established their roles as leading the discussion (moderator) or taking notes (observer). RF and JM have extensive experience of qualitative research. Focus groups lasted for between 58 and 72 min, with recordings being transcribed verbatim by an established agency. Transcripts were checked by RF/JM for accuracy. Field notes were collected during each focus group to facilitate analysis.

A Participant Information Sheet was provided; and informed written or verbally recorded consent obtained from each participant before the start of the focus group session. Participants were compensated for their time with a £25 shopping voucher, in accordance with the rates suggested by the National Institute of Health Research [43].

Participants were also invited to complete a brief demographic pro forma including four questions, based on the Family Affluence Scale [44].

Ethical approval was gained from the University of Hertfordshire Health, Science, Engineering & Technology Ethics Committee with Delegated Authority [protocol number: HSK/SF/UH/04840].

### 2.2 Analysis

Analysis was undertaken by RF, JM and LW using a thematic approach based on the stages offered by Braun and Clarke [45, 46] (Table 1). JM developed an initial codebook, once data saturation had been achieved; this was refined (combining, separating, and adding codes as necessary) and agreed with RF. Using a reflective approach, JM then developed draft themes and sub‐themes, which were discussed with RF and LW, alongside the field notes, to establish the final framework. Participants did not provide feedback on the findings.

**Table 1:** Summary of the analytical stages of the study.

Stages of data analysis (Braun and Clarke, 2006)	Application to this study
Becoming familiar with the data	The transcripts were read several times.
Generating initial codes	NVivo (version 12) was used, and this enabled a codebook to be developed.
Searching for themes	The codes were reviewed, and initial themes were generated.
Reviewing themes	The themes were re‐considered alongside the codes and raw data. Some themes were ‘collapsed’, leading to three main themes and six subthemes.
Defining and naming themes	The themes were named to reflect their content. Some of the participants' actual words are used to illustrate them and to further enable accurate reporting of the findings.
Producing the report	The content of the themes was presented, alongside illustrative quotes from participants.

### 2.3 Findings

In total, 19 participants with an age range of 18–44 years took part in a focus group. All were women who were trying to conceive (TTC), pregnant (Pt) or had babies under 6 months old (Table 2). Pregnant participants were in their second (*n* = 1) and third trimesters (*n* = 2). Participants and their partners were working, apart from P5 and P8 who did not specify working status.

**Table 2:** Demography of focus group participants (*n* = 19)^a^.

Demographic data	*n* (%)
Age, years	
18–24	3 (15.7)
24–34	9 (60.0)
35–44	7 (36.8)
Ethnicity	
White (British)	16 (5.2)
White (Irish)	2 (10.5)
Asian or Asian British	1 (5.3)
Country	
England	6 (31.6)
Wales	4 (21.1)
Scotland	4 (21.1)
Northern Ireland	5 (26.3)
Child < 6 months old, yes	15 (78.9)
TTC, yes	3 (15.7)
Pt, yes	3 (15.7)
Planning future pregnancy, yes	8 (42.1)
Number of children in household	
0	2 (10.5)
1	6 (31.6)
2+	11 (57.9)
Number of adults in household (mean, SD)	2.3 (0.8)
Family Affluence Scale (FAS) score (median, IQR) (higher score reflecting increased affluence)	6 (Range 3–8)

^a^ 19/19 (100.0%) FAS Score (0–8 points, where the higher score = increased affluence).

**Figure 1:**
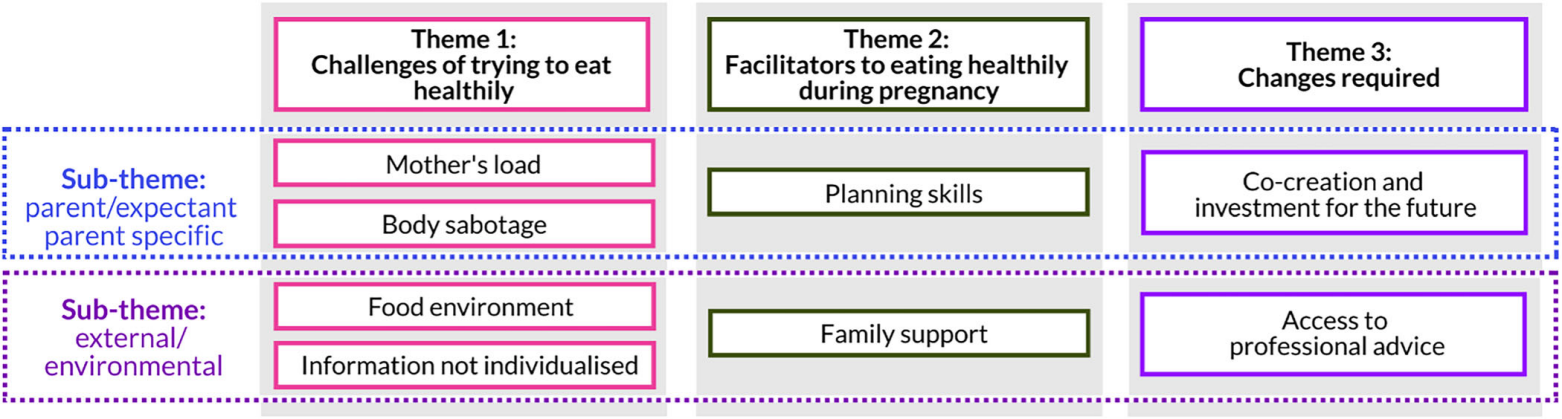
Themes and subthemes emerging from focus groups.

Analysis of the focus groups revealed three main themes and six subthemes (Figure 1). Subthemes were either parent/expectant parent specific or external/environmental.

### 2.4 Challenges of Trying to Eat Healthily

#### 2.4.1 Mothers' Load

All focus groups discussed the challenges of trying to eat a healthy diet across preconception and pregnancy. Participants commented on tiredness being compounded by the mother's load, for example, working full time and/or having a toddler, as being a barrier to eating as healthily as they would have liked. For example, P9 explained that although she ate well in her first pregnancy, during her second and third, she was too tired to eat:

When the second pregnancy I found it a bit harder to eat healthily because I was tired and I was working full time and had the baby, 1‐year‐old. I had them quite close together. So that was tough. And then the thirdpregnancy, I sort of gave up trying to eat healthily because I was exhausted, I think, a lot of the time with the other two. I was so busy with them*…*

(P9)

#### 2.4.2 Body Sabotage

Pregnancy side effects undermining the participants' ability to eat healthily was a topic of conversation in all focus groups. Despite their best intentions, participants found that they were not able to follow their healthy eating goals:

I thought I'd be all zen and be eating 20 vegetables at every meal, or whatever, and instead I was like, “Oh, no. I don't want to go in the kitchen.”

(P1)

One participant referred to this experience as the *‘*body sabotaging*’* (P17) their eating plans. Side effects such as heartburn when eating vegetables and salads and cravings for high‐carbohydrate foods such as doughnuts, biscuits, and cakes along with experiences of 'low nausea and not wanting to cook*’* (P17) and needing to ‘Close the door before I smell food*’* (P2).

P8 explained that they had hyperemesis gravidarum (excessive nausea and vomiting) and were in hospital having fluids *‘*because …even sort of taking in water was a struggle*’* (P8). She went on to say that whilst being hospitalised was difficult, the impact of not being able to follow the healthy eating advice was what she found *‘hardest’* to deal with.

#### 2.4.3 Food Environment

Participants defined eating healthily for pregnancy in terms of consuming fruit and vegetables. They identified the accessibility, availability, and cost in the food environment as being limiting factors to their consumption saying, *‘unhealthy food is cheaper and easier to buy and more accessible’* and so *‘*healthy eating in general is maybe not a priority*…*[for everyone]*’* (P10). However, P13 went on to explain that it was more than this, eating healthily might not be an option at all as money may simply not be available:

You think, “Am I going to just sacrifice having less healthy meals for the money?” Which is bad, really, because, obviously, you want to eat healthy, especially when you're pregnant. But sometimes, it's just not possible.

(P13)

Participants also commented on how the food environment appeared to encourage the consumption of unhealthy foods; P10 said:

I think just on how people shop and what we're sold on TV and in shops and convenience and all that definitely is affecting how people are eating.

(P10)

#### 2.4.4 Lack of Individualised Advice

There were mixed views about the adequacy of the information available from the NHS. It was felt to be straight forward and an important place to start, particularly in terms of foods to avoid. However, participants explained that the information lacked breadth, was *‘*quite generic about healthy eating*’* (P14) and was *‘*not quite the same as being empowered with lots of ideas of what would be good to eat*’*. (P1)

P16 explained that their midwife's response to being informed that they were vegetarian was to just *‘*write it down*’;* she commented that the midwife did not then follow this up with further questions or advice. For P1, who was vegan, the approach was different; she found that her midwife was more concerned about potential deficiencies of calcium and iron, implying that they were being *‘*reckless*’* (P1) with this dietary approach. P1 found that there was a tendency to take a medical stance and to prescribe iron supplements as opposed to a food‐focused approach:

*…*there didn't seem to be a lot of encouragement to try and sort it out through diet. It just seemed to be, “The easiest way is take this tablet.” Oh, you might be a bit constipated with it…. you can get constipated anyway during pregnancy, figure it out.

(P1)

Other participants suggested HCPS used shortcuts, relying on their experience, judgment, and visual assessment to decide if pregnant women needed further advice:

My midwife just looked at me and she was, like oh yes, you're not overweight like you're fine basically.

(P11)

While there was an expectation that midwives and GPs should be a source of nutritional advice, there was an awareness that they were not appropriately trained:

The midwives do so much, and there is so much that they have to do that adding nutrition onto that would just be so much more complex for what they're doing…sometimes maybe they are just a bit middle of the road, because they're afraid to give the wrong advice, because they don't know.

(P2)

### 2.5 Facilitators to eating healthily during pregnancy

#### 2.5.1 Skills in Food Provisioning

Skills in food provisioning such as planning and cooking skills were needed to ensure that the participants and their families ate a healthy diet. They found they needed to plan in advance take the opportunity to incorporate healthy food items wherever possible. One participant, who was TTC, noted that if they sliced and prepared fruit at the start of the day, they were much more likely to eat it. However, there was an acknowledgement that to be able to eat healthily there was a need ‘to be quite a good cook*’* (P11):

I'm 100% responsible for all of the food shopping and cooking and everything. My husband doesn't like cooking. I've got a 9‐week meal plan I've created just to take all the mental load out of it and the shopping list each week.

(P11)

However, possessing planning and cooking skills was not ubiquitous across the focus groups. The need for convenience was emphasised when participants were tired and lacked *‘*the enthusiasm to cook something healthy and nutritious’ (P3):

Convenience is a big thing. How quickly can it be cooked, preparation time, does it need to be chopped or peeled?…If I have to peel it and chop it up, maybe not*…*

(P10)

#### 2.5.2 Family Support

Participants also referred to the value of having partner and wider family support to help with meal preparation. While some said they did all the shopping as ‘[my partner] does not have the patience*’* (P19), others spoke of sharing the task:

1 week I'll maybe plan what we're going to have and the next my partner will. He would tend to do the food shop more than I would.

(P6)

However, others commented that having the meal prepared for them and support from the family ensured the consumption of a healthy diet:

I think having the meals prepared, and it being cooked for you… I was very lucky that I did actually live with my mum before we bought our house. So, I think that was definitely easier, just literally having healthy meals made for you, and put on the table, and someone to take the other child off you for a while.

(P18)

### 2.6 Changes Required

#### 2.6.1 Access to Professional Advice

Participants felt that all pre‐pregnancy and pregnant women needed to be empowered to be able to ensure that they ate a healthy diet. Their experiences were that HCPs assumed that if *‘*tests*’* were all fine and if this is ‘your second pregnancy*’* (P18) then no additional advice or guidance was needed. Participants felt that only those who appeared to be in need or who were eligible for benefits and at risk of not being able to access the right foods would be directed towards appropriate support.

They proposed that advice could be in the form of an app where *‘*professional advice*’* (P3) was available to *‘*set you up for the following few months*’* (P3) or from a nutritionist in a GP surgery that was ‘not just for people that are in a high‐risk group*’* (P9):

Why not have a nutritionist there that anybody can go and see at any time about any of their ailments? It should be something, “Right. You've been diagnosed with this. Okay, you want to see the nutritionist.” Even if it's just one appointment, “What are you eating? Here are a few small changes.” It doesn't need to be anything big, anything major. A couple of small changes that you can help in your general health.

(P2)

A ‘healthy eating course*’* (P18) in the form of group sessions was also suggested as part of the antenatal classes that many of the participants had experienced. This would give additional context to the information given by the midwife in the one‐to‐one appointment.

There was a view that the one dimensional *‘*do not eat*’* (P16) instruction was *‘*disempowering*’* (P1) and a facilitatory practical approach on *‘*how to cook a meal*’* (P16) was proposed. There appeared to be a lack of information for women who were trying to conceive, particularly those who were overweight, and which was individualised:

*…*I just feel like some women really struggle to conceive and if there's a potential that some really tailored advice on their diet could help then I think that would be very welcome and could potentially save money further down the line.

(P3)

#### 2.6.2 Co‐Creation and Making the Case for Investment for the Future

Participants suggested that the information could be made more relevant if it was developed with pre‐pregnancy and pregnant women to ensure it was useable and relevant for them:

I think that sometimes it, sort of, feels like you've got to do a lot of reading, and almost become an expert in something before you can have an opinion. I think a lot of the resources and stuff could be rewritten so they're almost user‐friendly, rather than a little bit like a teacher's handout. You could do focus groups, or all sorts of things to help create stuff that was just really useful.

(P1)

Participants emphasised the need for support for pre‐pregnant and pregnant women to ensure the ongoing health of the population:

[to] ensure that there's enough in the workforce to offer this sort of support, you know, directly to pre‐pregnant and pregnant women.

(P9)

[because] you've got someone else, depending on you as well and they're kind of the future of our country, so sounds like a good investment to me*…*

(P3)

Participants believed that access to information on healthy eating during pregnancy should be available to all, not just those who had been identified as high risk. They felt that additional support was a government responsibility, as this would benefit all. The advice should be facilitatory and individualised, co‐created with pregnant women to avoid the advice being instructional and generic. The proposed formats were for something ‘tangible*’* (P12) and accessible to all. Access did not necessarily need to be via HCPs, but could include other options such as online apps, group sessions (as part of antenatal courses) and/or one‐to‐one advice in GP practices from nutritionists or dietitians.

## 3 Discussion

This study sought to provide women's perspectives of nutrition during pre‐conception and pregnancy. Challenges, facilitators, and changes needed were discussed, revealing specific themes relating to parents/expectant parents and to external influencers.

Participants were motivated to consume a healthy diet. However, the pregnancy itself, plus the need to juggle their responsibilities with work and other children, resulted in exhaustion and made consuming a healthy diet challenging. Research suggests a need to prioritise either healthy eating and/or parenting [31]. In our study, some participants showed surprise at the impact of pregnancy, having expected to be able to cope with this additional life change, and appeared frustrated with not being able to follow their goals. Side effects of pregnancy have also been found by other researchers to be a barrier to healthy eating for the participants which can result in a diet of lower nutritional value than intended [25, 31, 47, 48, 49, 50, 51]. Other studies suggest that focusing on healthy eating, once symptoms have eased and when there is likely to be an increase in connectedness with the baby, may result in improved outcomes [50].

Accessibility, availability, and cost of healthy foods were found to be barriers to a healthy diet; other studies concur that healthy meals are expensive [31], and that cost can be a barrier [47, 52]. In our study, there was some evidence of access and availability being a barrier. Interruptions to the food system due to COVID‐19 may have impacted on the variety of fresh produce in supermarkets [53] and so may also have played a role.

Although participants were able to access information from the NHS and were able to apply this to foods to avoid, the advice was considered basic and not applicable to those whose dietary approach required additional planning. They expected to receive individualised advice from their HCP. Previous studies support this finding. Garcia et al. [49] and Abayomi et al. [27] both found that nutrition information provided by midwives was considered basic. However, Garcia et al. [49] also identified that information was judgemental, and Wise [47] reported that pregnant adolescents needed guidance on how to contextualise information. McCann et al. [28] propose that midwives need clinical leadership to improve their role in delivering nutritional advice to pregnant women. Swift, Langley‐Evans, Pearce et al. [54] suggested that pregnant women in the UK had a low awareness of guidance on diet in pregnancy, even if attending antenatal appointments, and commented that individual pregnancy experiences necessitate tailored advice.

Participants felt that HCPs assumed that they did not need dietary advice, either due to ‘looking healthy’ or carrying a second pregnancy. Similarly, studies have found that if women had been pregnant before, they received limited/no information, based on the assumption that they had existing knowledge [25, 26, 50] with midwives defaulting to written information if they perceived a mother to be healthy [26].

Participants felt that midwives took a medical approach to nutrition, focusing on food safety issues and potential vitamin and mineral deficiencies, as opposed to discussing healthy eating overall. Participants expressed a desire for less focus on what not to eat; instead, they needed information on how to eat healthily. Arrish et al. [26] also identified a medical approach used by midwives and noted a lack of healthy eating guidance in written information. However, while the focus on suspected deficiencies was not necessarily welcomed by participants in our study, research suggests there is a lack of knowledge by parents [25] and evidence of low intake of iron and calcium [5]. Studies have suggested a facilitatory approach to nutrition in pregnancy to help empower women [25, 48, 55]. For example, van Lonkhuijzen et al. [55] undertook an RCT comparing the impact of additional facilitatory and reflective nutrition consultations with midwives and dietitians and standard care; they found a positive influence on dietary habits amongst pregnant women.

Planning skills were found to be essential to support a healthy diet and to ensure that the family were fed. Garcia et al. [49] found that meal planning, buying healthy ingredients in bulk and freezing them were helpful strategies. Another facilitator was access to healthy convenience foods and recipes that required little preparation and cooking time. Research has identified the value of convenience foods [50], quick, easy recipes with staple ingredients [49] and having healthy snacks (such as fruit) readily available [52], this was also highlighted in our study, along with key skills such as being able to cook or drive to the supermarket [47].

Having a partner and or family help to prepare meals was beneficial and is reported in the literature [31, 47, 56, 57]. For example, Wise [47] found that family support for adolescents improved nutritional intake. Rhodes et al. [57] undertook a qualitative study with men and women in the last trimester of pregnancy or with a child under the age of 18 months; they found that the level of support from partners was variable, some were able to provide full emotional and practical support (such as preparing meals with vegetables), others felt their role was to help with choices/cravings. The authors concluded that nutrition education should include partners. This finding was echoed in a study by Quayyum and Dombrowski [58], whereby women felt it imperative that their partners joined them in trying to eat healthily as a form of support and encouragement. Overall, social systems (partner, family, and friends) were regarded as important.

Participants indicated their preference for nutritional advice to be made available in primary care from HCPs who specialise in nutrition such as a nutritionist or dietitian, however few had seen one. Similarly, Misita et al. [30] found that out of 100 women, 90% had not seen a dietitian during pregnancy but 48% would like to; Wise [47] stated a need for advice to be available in person; Super and Wagemakers [48] highlighted a wish for personalised advice that considered preferences, culture and personal situation. Our participants felt that individualised nutrition information should be made available to all. Although the provision of advice in pregnancy by a dietitian has been found to improve nutritional outcomes [59], this is often prioritised to those who have complications [30, 48]. Super and Wagemakers [48] reported that personalised advice from dietitians was not available to pregnant women unless they had complex issues or were following a special diet such as ‘gluten free’. NICE [60] only mention referral to a dietitian for those pregnant women with a pre‐pregnancy body mass index (BMI) over 30 kgm^2^ and NICE [5] highlight that if there is a BMI of over 40 kgm^2^ at the booking appointment, a referral to a specialist obesity service is recommended. To combat this issue, Super et al. [29] propose that dietitians and midwives should collaborate to provide nutritional advice and Abayomi et al. [27] suggest that dietitians are needed to provide the training to midwives to support this collaboration.

Participants also proposed alternative ideas to the one‐to‐one standard advice format such as an app that provides professionally developed nutritional advice. Studies have found a preference for [27] and satisfaction with pregnancy nutrition apps [61] and a positive impact on adherence to dietary recommendations [62]. However, although they may help with health literacy [22, 61], apps may need additional regulation [56] and it is unclear whether their use can have a positive impact on nutrition outcomes in pregnancy [63]. A healthy eating or cooking course as part of antenatal care, suggested by participants, has also been identified by others [47, 64, 65]. However, consideration should also be given to drop‐in sessions as opposed to a structured or formal intervention as some families may prefer this [18, 21]. In a recent qualitative study (UK and Ireland) exploring cooking programmes during pregnancy, participants identified value in leveraging digital technologies, potentially combined with in‐person services and supported by the health service [65]. Whilst Irish participants preferred online delivery, UK participants preferred in person or hybrid programmes, suggesting regional differences [65].

Participants in our study made the case for co‐creation of nutrition information resources as this would ensure relevance and a format that met the needs of end users. There has been some research involving pregnant women in service design [27] and in information design where the development of written information improved quality and usability [66, 67].

The participants were motivated to eat healthily but were limited by other priorities. The impact of pregnancy on everyday lives was far reaching. Some participants were working full time, coping with pregnancy side effects, managing children against the backdrop of an increased imperative to eat healthily. To be able to pursue a healthy diet our participants needed to be motivated, resourceful and utilise extensive planning and cooking skills to meet their nutritional goals. Despite all the challenges of having a healthy diet, participants returned to planning for, or being pregnant, as a valuable opportunity for HCPs to promote healthy eating. They felt that it appeared that this was missing from the services they had experienced, and that Government had a responsibility to promote it.

Finally, while challenges and facilitators were presented in the findings as separate themes, it is acknowledged that there will be an overlap and connection between these themes. For example, where a participant experienced a challenge in not having access to support or possessing skills to ensure a healthy diet during pregnancy, another participant may have had access to that support or possess those specific skills.

### 3.1 Limitations

Participants were recruited from across all 4 UK nations, giving a broad non‐localised view on the experiences of pre‐pregnant/pregnant women trying to eat healthily. However, our sample was limited by the lack of diversity in terms of ethnicity and socioeconomic status so will not have been representative of all parents/expectant parents and generalisations cannot be made. As there are different services available across the UK, participants will have had varying experiences and, in addition, there was not a specific aim to replicate different socioeconomic or cultural groups. Our recruitment was from private Facebook groups and so pregnant women who were not members of these groups were excluded from taking part. Further research could focus on exploring, in more detail, experiences in each of the four nations, as well as different socioeconomic backgrounds and cultural groups; this would enable comparison of the different service approaches and policies across the UK. Nevertheless, our study provided a unique and invaluable insight.

Participants may not have felt that they could speak freely in the focus groups, particularly if they did not share demographic characteristics with others. However, the moderators were highly experienced and utilised frequent encouraging phrases to enable participants to feel empowered to take part in discussions.

The focus groups were run by two dietitians. Although neither were working in antenatal care, participants may have felt that they could not speak freely about their diet for the fear of being judged. They may have expected to receive nutritional advice; however, none asked for any and provision was made for GP referral if it should have been needed. While the setting for the focus groups necessitated them being held online, we do not know how responses may have been different if they had been held face to face. Qualitative research is subjective, so it was important to ensure that maximum rigour was applied; the team engaged in ‘reflexivity’ at all stages of the study and held regular meetings with The Food Foundation who provided an external critical and nonacademic perspective.

## 4 Conclusion and Recommendations for Practice

Despite widespread agreement on the importance of women eating a healthy diet during pre‐conception and pregnancy, recommendations from government and nongovernment bodies, and willingness from those women affected, in practice many barriers remain in place. There are several ways in which provision of nutrition advice could be made more fit for purpose. There is potential to link a range of resources relating to antenatal care, empowering and educating midwives to deliver individualised advice and/or to refer to other sources of information such as apps and privately run antenatal classes. As improvements in nutritional intake may not be possible in the early stages of pregnancy, consideration should be given to the impact of healthy eating throughout pregnancy, nutrition practices in subsequent pregnancies and during childhood as well as the role of partners and support systems in underpinning women's daily life and dietary practices during pregnancies. There are opportunities to join up nutrition and dietetic services with midwifery and health visiting ones to maximise this.

Nutrition information should consider the impact of the ‘mother's load’ on nutritional intake. Making information available to pre‐pregnancy and pregnant parents that focuses on healthy convenience foods and snacks, quick easy recipes, meal planning, and development of cooking skills may be more beneficial than concentrating on maternal food safety aspects. Involving parents in the co‐creation of resources may enable their relevancy and impact.

## Author Contributions

**Jane McClinchy:** conceptualization (equal), formal analysis (lead), funding acquisition (equal), investigation (equal), methodology (equal), writing – original draft (lead), review and editing (equal). **Rosalind Fallaize:** conceptualization (equal), formal analysis (equal), funding acquisition (equal), investigation (lead), methodology (equal), visualization (lead), writing – review and editing (equal). **Kelly Parsons:** conceptualization (equal), funding acquisition (equal), methodology (equal), writing – review and editing (equal). **Lisa Whiting:** conceptualization (equal), funding acquisition (lead), methodology (equal), project administration (lead), resources (lead), writing – review and editing (equal).

## Ethics Statement

Ethical approval was gained from the University of Hertfordshire Health, Science, Engineering & Technology Ethics Committee with Delegated Authority [protocol number: HSK/SF/UH/04840].

## Conflicts of Interest

The authors declare no conflicts of interest.

## 1 Peer Review

The peer review history for this article is available at https://www.webofscience.com/api/gateway/wos/peer-review/10.1111/jhn.70122.

## 2 Transparency Declaration

The lead author affirms that this manuscript is an honest, accurate, and transparent account of the study being reported. The reporting of this study is compliant with COREQ [31]. The lead author affirms that no important aspects of the study have been omitted.

## Data Availability

The data that support the findings of this study are available on request from the corresponding author. The data are not publicly available due to privacy or ethical restrictions.
